# Rationale and design of the Measuring Athlete’s Risk of Cardiovascular events (MARC) study: The role of coronary CT in the cardiovascular evaluation of middle-aged sportsmen

**DOI:** 10.1007/s12471-014-0630-0

**Published:** 2014-11-20

**Authors:** T. L. Braber, A. Mosterd, N. H. J. Prakken, P. A. F. M. Doevendans, W. P. Th. M. Mali, F. J. G. Backx, D. E. Grobbee, R. Rienks, H. M. Nathoe, M. L. Bots, B. K. Velthuis

**Affiliations:** 1 Department of Radiology, University Medical Center Utrecht, Utrecht, the Netherlands; 2 Department of Cardiology, Meander Medical Center, Amersfoort, the Netherlands; 3 Department of Cardiology, University Medical Center Utrecht, Utrecht, the Netherlands; 4 Department of Radiology, University Medical Center, Groningen, the Netherlands; 5 Department of Rehabilitation, Nursing Science and Sport, University Medical Center Utrecht, Utrecht, the Netherlands; 6 University Medical Center Utrecht - Julius Centre for Health Sciences and Primary Care, Utrecht, the Netherlands; 7 University Medical Center Utrecht, Heidelberglaan 100, Room E01.132, PO Box 85500, 3508 GA Utrecht, the Netherlands

**Keywords:** Athletes, Coronary artery disease, Risk, Coronary artery calcium score, Multidetector computed tomography

## Abstract

**Background:**

More than 90 % of exercise-related cardiac arrests occur in men, predominantly those aged 45 years and older with coronary artery disease (CAD) as the main cause. The current sports medical evaluation (SME) of middle-aged recreational athletes consists of a medical history, physical examination, and resting and exercise electrocardiography. Coronary CT (CCT) provides a minimally invasive low radiation dose opportunity to image the coronary arteries. We present the study protocol of the Measuring Athlete’s Risk of Cardiovascular events (MARC) study. MARC aims to assess the additional value of CCT to a routine SME in asymptomatic sportsmen ≥45 years without known CAD.

**Design:**

MARC is a prospective study of 300 asymptomatic sportsmen ≥45 years who will undergo CCT if the SME does not reveal any cardiac abnormalities. The prevalence and determinants of CAD (coronary artery calcium score ≥100 Agatston Units (AU) or ≥50 % luminal stenosis) will be reported. The number needed to screen to prevent the occurrence of one cardiovascular event in the next 5 years, conditional to adequate treatment, will be estimated.

**Discussion:**

We aim to determine the prevalence and severity of CAD and the additional value of CCT in asymptomatic middle-aged (≥45 years) sportsmen whose routine SME revealed no cardiac abnormalities.

**Electronic supplementary material:**

The online version of this article (doi:10.1007/s12471-014-0630-0) contains supplementary material, which is available to authorized users.

## Background

### Screening athletes to prevent cardiovascular events

Sudden cardiac death is often the first manifestation of coronary artery disease (CAD), and is responsible for ≈50 % of the mortality of cardiovascular disease in developed countries [1]. Regular physical exercise is recommended to reduce cardiovascular morbidity and mortality and is gaining increased popularity, especially in middle-aged and older persons [2]. Yet, exercise transiently increases the risk of cardiovascular events, particularly in those with unknown cardiac disease. More than 90 % of acute exercise-related cardiac events occur in men, predominantly those aged 45 years and over [3]. While sudden cardiac death in younger athletes (35 years or younger) is mainly caused by cardiomyopathies, electrical heart disease and coronary anomalies, in older athletes it is predominantly caused by CAD (80 %) [4].

A recent paper on cardiac arrest during long distance running implicated a causal role for demand ischaemia in athletes with (unknown) CAD [2]. Absence of coronary plaque rupture in these persons was surprising because prior data [5] and expert consensus documents [4] have suggested that exercise-induced acute coronary events result from atherosclerotic plaque disruption and coronary thrombosis. It follows that early identification of CAD should be an important goal in the pre-participation evaluation of middle-aged persons.

Despite the rare occurrence of cardiac events in young athletes (<2 per 100,000 athletes per year) the study group of Sport Cardiology of the European Society of Cardiology (ESC) recommends mandatory pre-participation screening (based on medical history, physical examination and a resting electrocardiogram (ECG)) of athletes aged 35 years or younger [6]. Directing screening efforts at the rapidly growing group of older athletes who have a 10-fold higher risk of exercise-related cardiac arrests [3]—mainly attributable to CAD—should be considered as well [7].

The 2011 ESC position paper on cardiovascular evaluation of middle-aged/senior individuals engaged in leisure time sports activities advocates the use of maximal exercise testing. This is now frequently performed in the course of a sports medical examination (SME), in addition to medical history, physical examination, and the resting ECG [8].

### Cardiovascular event risk: exercise testing and cardiac imaging to detect coronary artery disease

Traditionally, risk scores such as the ESC Systematic COronary Risk Evaluation (SCORE) are used to estimate cardiovascular event risk in asymptomatic persons, aiming to divide them into low, intermediate and high risk categories. The SCORE risk categories correspond to 0–4 %, 5–9 % and 10 % or higher 10-year cardiovascular mortality risk [9]. Cardiovascular risk scores have not specifically been developed for persons who are physically active. As exercise favourably influences cardiovascular risk factors, e.g. by reducing weight and blood pressure and improving the lipid profile, traditional cardiovascular risk scores may underestimate CV risk in persons who exercise regularly [10]. It has also been suggested that regular, intense physical exercise (e.g. multiple marathon running) in itself may be harmful to the heart by causing myocardial fibrosis [11] and by contributing to coronary artery plaque formation with cardiovascular events occurring at an alarmingly high rate (21 % 10-year event rate) in a group of 108 recreational German marathon runners aged 50 years or older [10]. Cardiovascular events exclusively occurred in those with a coronary calcium score (CACS) higher than 100 Agatston units (AU).

Adding maximal exercise testing to the cardiovascular evaluation of middle-aged/senior athletes, as advocated by the ESC, may be useful to identify some persons at high risk, by detecting a physiologically significant coronary artery stenosis [12]. Several studies have indicated that asymptomatic men with an abnormal exercise test received the greatest benefits of interventions to reduce risk factors [13]. Notwithstanding the low predictive value (high false-negative rate) for CAD of both resting and exercise electrocardiography in asymptomatic individuals, exercise testing is still frequently performed in the course of a sports medical check-up [14]. This is partly attributable to the fact that the results of exercise testing will also provide information on cardiorespiratory fitness relevant to recommendations for subsequent training programs.

Coronary CT (CCT) can be of added value by providing a minimally invasive opportunity to image the coronary arteries for coronary artery calcium (CAC), atherosclerotic burden and coronary stenosis [15].

Non-contrast CCT for the assessment of CAC provides a non-invasive direct measure of burden of coronary atherosclerosis and is an independent predictor of cardiovascular events [16]. The CACS is the most powerful cardiac risk prognosticator in the asymptomatic population, with consistent superiority to all risk factor-based scores [17]. Absence of CAC is associated with a very low risk of future cardiovascular events in asymptomatic as well as symptomatic individuals [18]. The amount of CAC, however, relates poorly to the degree of luminal narrowing of the coronary arteries and a low CACS does not exclude CAD [19, 20]. The ‘Agatston Score’ remains the most commonly used measure to quantify CAC because most previous large-scale trials and cross-sectional studies used this method, so that large reference datasets exist (e.g. http://www.mesa-nhlbi.org/Calcium) [21]. Coronary artery calcification is age, gender, and race dependent. Agatston scores are often classified into five groups according to severity as listed in Table 1 [22]. A score <100 Agatston units (AU) is ranked as low risk, scores between 100 and 400 AU as intermediate (moderate) and above 400 AU as severe risk of future atherosclerotic cardiovascular events [22]. CACS is increasingly used as a tool to expand risk stratification in asymptomatic persons with an intermediate risk of cardiovascular events [23]. The low number needed to screen in the general population to identify an individual with moderate or severe CAC (8 and 20 respectively) seems to provide a justification for extending CAC testing to lower risk individuals [16] as CAC is also associated with risk of cardiovascular events among individuals with few or no risk factors. The 5-year number needed to treat to prevent a cardiovascular event in persons with a CACS ≥100 AU is low (19) [24] compared with the 5-year number needed to treat to prevent a cardiovascular event in patients with mild to moderate (140–160 mmHg) hypertension (122 to 135) [25].

**Table 1:** Classification of coronary calcium score

Absolute value (Agatston units)	Ranking
0	Absent
>0 < 10	Minimal
≥10 < 100	Mild
≥100 < 400	Moderate
≥400 < 1000	Severe
≥1000	Extensive

Classification of coronary calcium absolute content evaluated by cardiac CT and quantified by Agatston units

### Coronary CT angiography

Low-dose coronary CT angiography (CCTA) can visualise, qualify (calcified, non-calcified, mixed plaques), and quantify (total atherosclerotic burden, degree of luminal stenosis) CAD and is increasingly used to rule out CAD in persons presenting with chest pain. The diagnostic accuracy of CCTA is high, regardless of risk profile [26]. In the near future CT fractional flow reserve is likely to provide information on the functional significance of coronary artery stenosis [27]. The vulnerable plaque paradigm states that plaque composition is a better predictor of acute coronary events than stenosis grade [28]. As athletes normally have a lower heart rate and BMI, they are suitable candidates for low radiation dose (2–3 millisievert—mSv) CCTA to determine the presence and extent of CAD. This dose corresponds to the yearly background radiation each person receives in Europe [29]. Furthermore, a recent position paper that analysed acute and long-term composite risks related to cardiovascular imaging showed that life-time risk of imaging procedures for fatal events is small compared with the general risk of fatal cardiac events by CAD in both asymptomatic and symptomatic populations [30].

### Aim

The aim of the Measuring Athlete’s Risk of Cardiovascular events (MARC) study is to determine the prevalence and severity of CAD and the additional value of CCT in asymptomatic middle-aged (≥45 years) sportsmen whose routine SME revealed no cardiac abnormalities.

## Methods/design

MARC is a prospective, cross-sectional study. Asymptomatic sportsmen aged 45 years and older, without known cardiovascular disease, engaged in competitive or recreational leisure sports or contemplating initiating regular exercise and with a normal SME (including resting and exercise electrocardiography) will be invited to undergo additional CCT. Baseline characteristics, including demography, cardiovascular risk factors, sports history, medical history and medication use, will be obtained. The results of the CCT, combined with the conventional cardiovascular risk profile, will be used to provide the participants with a tailored cardiovascular advice, based on the prevailing cardiovascular disease prevention and sports medicine guidelines [9, 31].

Based on the number of participants (*n* = 108) in the only cardiac CT study of asymptomatic athletes to date and the yearly number of persons (*n* = 300–350) performing a SME at an average Dutch sports medical department who would qualify for participation in MARC, we aim to include 300 asymptomatic participants to obtain estimates of the prevalence of coronary artery disease with reasonable confidence intervals and to determine the added value of CCT (CACS and CCTA) in the prevention of cardiovascular events. Given the 10- to 30-fold higher incidence of exercise-related cardiac arrests in men than in women we choose to enrol men only [32].

### Coronary CT imaging

Coronary CT is performed using a 256-slice CT scanner (Philips Healthcare, Best, the Netherlands). A non-contrast coronary CT is acquired first to calculate the CACS (scan parameters 120 kV, 60 mAs). Participants with a heart rate >65 beats/min will receive 5 to 20 mg metoprolol (Selokeen AstraZeneca, Zoetermeer, the Netherlands) intravenously before CCTA. All participants receive two puffs of sublingual nitroglycerine spray just before the CCTA. CCTA scan parameters will be as follows: 120 kV; 210 mAs; 90–120 ml (depending on weight) non-ionic contrast material (Iopromide, 300 mg I/ml; Ultravist, Bayer Healthcare, Berlin, Germany) will be injected at a speed of 6–6.7 ml/s followed by 30–40 ml saline injected at the same speed. The CCTA is acquired with prospective ECG triggering at a mid-diastolic phase (78 %).

To determine the amount and distribution of soft and mixed coronary artery plaques CCTA will be done regardless of the CACS. In clinical practice CCTA is often withheld in patients with a CACS >400 AU because blooming artefacts might hinder the evaluation of coronary artery stenosis [33].

The total CCT radiation dose to which participants will be exposed is anticipated to be 3 mSv on average [34]. Participants receive extensive written and oral information prior to examination. If renal function is unknown, the creatinine is measured before the scan. Participants with renal dysfunction (estimated glomerular filtration rate <60 ml/min/1.73 m^2^) will be excluded from the study. Blood pressure and heart rate are available from the SME and will also be measured before the CT scan.

### Image analysis

CT scans are sent to a workstation (IntelliSpace Portal, Philips Healthcare) for processing by experienced technicians. All CT scans are analysed by one of two experienced observers.

### Assessment of coronary artery calcium score

CACS is measured on the non-contrast CT with the Agatston scoring method [35]. Coronary artery calcium is measurable above a density of >130 Hounsfield units (HU) in a coronary artery. Total CACS is calculated by the sum of all lesions in all four coronary arteries and their side branches. The total Agatston score will be compared with the MESA database [21].

### Assessment of coronary CT angiography

Specialised technicians use semi-automated vessel analysis to make multiple curved multiplanar reconstructions (MPR) of all coronary arteries on the CCTA data. The radiologist views these MPR reconstructions as well as the thin-slice CCTA source images with interactive maximum intensity projection and MPR viewing possibilities in all planes. Image quality, plaque characteristics and coronary lumen stenosis will be analysed on a 16-segment basis according to the modified American Heart Association classification [36].

Plaque composition will be evaluated in a qualitative manner as calcified (more than 50 % of the plaque area calcified), mixed (plaques with <50 % calcium) and non-calcified (plaques without calcium) [37].

Total atherosclerotic plaque burden will be measured with both the segmental involvement score (SIS) and the segment stenosis score (SSS) based on the 16-segment coronary artery model. [38] Luminal stenosis will be graded as absent, minimal (1–24 %), mild (25–49 %), moderate (50–69 %), and severe (≥70 %) [39] narrowing on the basis of diameter measurements comparing the diameters of the maximal stenosis to a reference diameter proximal and distal to the stenotic area. If severe calcifications are present and quantification of stenosis is difficult, the radiologist will refrain from stenosis quantification and score the segments involved as ‘calcified, stenosis unclear’.

### Outcome and consensus meetings

The primary outcome is the presence of relevant CAD that will be defined as one or more of the following on CT: a CACS ≥100 AU or luminal stenosis ≥50 %. Participants with a CACS ≥100 AU will be reclassified to high risk [24]. Consensus meetings with a panel of at least two (sports) cardiologists and one radiologist will be held regularly to advise on the management of participants found to have relevant CAD. As a general rule participants with a CACS between 100 and 400 AU without obstructive CAD will be recommended to initiate treatment with statins, in addition to lifestyle advice [40]. Participants with a CACS ≥400 AU or coronary stenosis ≥50 % will be given the opportunity to consult a cardiologist for further cardiovascular workup.

### Statistical analysis

Analysis of the (baseline) results from this study will be descriptive (presence and extent of CAD). Descriptive statistics will be presented as means and standard deviations for continuous variables, and frequencies and percentages for categorical data. The two-tailed independent *t*-test will be used for comparison between groups (e.g. those with and without coronary artery disease) and analysis of variance will be used to compare means of more than two groups. The chi-square test will be used to examine the association between categorical variables. Linear regression and multivariate regression analysis will be used to study the relation between characteristics of the participants and CCT determined CAD.

The number needed to screen to prevent one cardiovascular event (defined as coronary heart disease events -myocardial infarction, death from coronary heart disease, definite angina, probable angina resulting in revascularisation or resuscitated cardiac arrest-, stroke, or other atherosclerotic death or other cardiovascular death) within 5 years will be computed based on the reciprocal of the prevalence of CAD (CACS ≥100 AU and / or luminal narrowing ≥50 %) (multiplied by 100) multiplied by the number needed to treat to prevent one cardiovascular event [24].

Statistical analyses will be performed with SPSS version 22.0 (SPSS Inc., Chicago, Illinois, USA). A p-value of less than 0.05 will be regarded as statistically significant.

### Ethical considerations

This study has been approved by the regional Medical Ethics Committee (VCMO, Nieuwegein, the Netherlands) and subsequently approved by the local ethics board committee of the University Medical Center Utrecht, the Netherlands. The study is being conducted according to the principles of the Declaration of Helsinki and in accordance with the Dutch Medical Research Involving Human Subjects Act. Participants are only included in the MARC study after written informed consent is obtained.

## Discussion

The MARC study will assess the additional value of low-dose coronary CCT to determine the prevalence and severity of CAD in 300 asymptomatic middle-aged (≥45 years) sportsmen, in whom an SME found no cardiac abnormalities. Participants of the MARC study should reflect the fast growing group of men aged 45 years or older who decide to undergo a SME that includes exercise testing. As such, we anticipate to include both competitive and recreational athletes. The majority of them are likely to be engaged in high dynamic high static sports (e.g. race cycling) and high dynamic low static sports (e.g. long distance runners). The number needed to screen with CCT to prevent the occurrence of one cardiovascular event in the next 5 years will be estimated.

Sudden cardiac death is the most devastating sport-related event. Although regular exercise reduces the risk of cardiovascular disease, the risk of an acute cardiac event is transiently increased during and immediately after vigorous exercise [4] Sports-related cardiac events occur mainly in those with unknown cardiac disease, which prompts many sportsmen to undergo a SME with exercise testing. The MARC study offers the most comprehensive cardiovascular evaluation of middle-aged sportsmen to date, by adding CCT (both CACS and CCTA) to the routine SME.

CT scanning is generally considered safe but, apart from radiation exposure (discussed in the Background section: CCTA), the following considerations should be taken into account when evaluating asymptomatic persons with CCT. Potential risks of CCTA include adverse reaction to the contrast media (nausea, skin hives in 1 to 3 % of participants, fatal adverse reactions in 1 in 100,000 participants) and contrast media-induced renal insufficiency (contrast nephropathy). The occurrence of contrast nephropathy is directly related to renal function; by selecting participants with a GFR higher than 60 ml/min contrast nephropathy is unlikely to occur [41].

The result of the CCT may lead to additional diagnostic tests with extra costs and risks not covered by this study. However, the Eisner study demonstrated that CAC scanning compared with no scanning in asymptomatic volunteers can improve cardiac management without incurring a significant increase in downstream medical costs [42]. Incidental findings will be reported to the participant and his general practitioner. Pulmonary nodules are likely to be common incidental findings in our study and will be managed according to the Fleischner criteria [43].

The results of the MARC study will give insight into the added value of CCT in the cardiovascular evaluation of asymptomatic sportsmen aged 45 years and older who have undergone a routine SME. We anticipate that our study will provide guidance in the debate on the optimal pre-participation evaluation of the rapidly growing group of older sportsmen in whom CAD is the main cause of cardiovascular events.

## Electronic supplementary material

Below is the link to the electronic supplementary material.

ESM 1 (PDF 175 kb)

ESM 2 (PDF 136 kb)
