# Supplementary material for: Rationale and design of the Measuring Athlete’s Risk of Cardiovascular events (MARC) study: The role of coronary CT in the cardiovascular evaluation of middle-aged sportsmen
Source: Neth Heart J. 2014 Nov 20;23(2):133–8. doi: 10.1007/s12471-014-0630-0 (PMC4315792; doi:10.1007/s12471-014-0630-0)
Supplement: Supplementary file 1 — (PDF 175 kb) [file 12471_2014_630_MOESM1_ESM.pdf]

Verenigde Commissies Mensgebonden Onderzoek

VCMO

VCMO
Deelnemende instellingen:

St. Antonius Ziekenhuis Nieuwegein/Utrecht
Diakonessenhuis Utrecht/Zeist
Meander Medisch Centrum Amersfoort/Baarn
Onze Lieve Vrouwe Gasthuis Amsterdam

Secretariaat:
Koekoekslaan 1
3435 CM Nieuwegein

Postbus 2500
3430 EM Nieuwegein

tel.: 030-6093580
030-6094420
fax: 030-6094419
e-mail: info@vcmo.nl
internet: www.vcmo.nl

Meander Medisch Centrum
T.a.v. dr. A. Mosterd, cardioloog
Postbus 1502
3800 BM AMERSFOORT

Kenmerk: V.11.416/R-11.44M/cl — Datum: 25-11-2011

Betreft: commentaar commissie NL38234.100.11
R-11.44M/MARC

Geachte heer Mosterd,

Uw studie met titel: **"Measuring Athlete's Risk of Cardiovascular events"**, VCMO registratienummer **R-11.44M** en acroniem **MARC**, is door de commissie besproken tijdens haar vergadering van 07-11-2011.

Uit deze vergadering zijn de volgende opmerkingen gekomen:
<u>Algemeen</u>
De commissie is van mening dat het een wetenschappelijk relevante studie betreft. Echter, het protocol bevat geen inschatting van de onderzoekers in hoeverre MDCT plotse dood bij sporters zal voorkomen.

<u>Methodologie</u>
De commissie is van mening dat de studie goed is opgezet.

1. Het is een nuttig vooronderzoek hoewel het onderzoek nog geen direct antwoord geeft op de vraag of CT scan informatie helpt bij het voorspellen van plotse dood bij sporten.
2. Er wordt begrijpelijkerwijs geen gouden standaard onderzoek verricht (coronairangiografie), maar het wordt nu als gouden standaard gepresenteerd. De klinische betekenis is beperkt als er geen follow-up wordt gedaan.
3. Aangegeven wordt dat voor de analyse een net reclassification index wordt berekend, het is echter wat onduidelijk hoe. De commissie ziet dit graag verduidelijkt.

<u>Vraagstelling</u>

4. De vraag die uit het ABR-formulier naar voren komt is tweeledig: (1) zijn hartproblemen beter op te sporen? en (2) leidt dit tot minder hartproblemen in de toekomst? Op pag. 4 van het protocol staat dat men de prevalentie en de ernst van CAD wil bepalen in deze groep. En als secondary objective dat men de relatie tussen risicofactoren en CAD en tussen lichamelijke activiteit en CAD wil bepalen. Het is daarom niet helemaal duidelijk wat de vraagstelling is. De commissie ziet dit graag verduidelijkt en geüniformeerd in de diverse documenten.

Medisch

5. De commissie is van mening dat ook als baseline ergometrie aanwezig moet zijn en dat eveneens baseline echocardiografie (inclusief linker ventrikel ejectiefractiebepaling) moet worden verricht, teneinde ook niet-ischemische hartafwijkingen op te sporen. Het protocol moet hier op worden aangepast.

De commissie ziet, naast de voordelen van deze studie, de volgende beperkingen.

6. De screening is niet van toegevoegde waarde bij laag en hoogrisico patiënten. Dit is ook beschreven in het studieprotocol. Personen met dit risicoprofiel zouden dan ook uitgesloten moeten worden van deelname aan de studie. De studie zou personen met een intermediair risico moeten includeren.
7. Nut van medicamenteuze behandeling van asymptomatische patiënten op klinisch beloop is niet bewezen. Graag ook verduidelijken in de patiënteninformatiebrief.
8. Potentieel nadelige gevolgen ten gevolge van vals positieve testen en blootstelling aan straling (vooral bij herhaalde MDCT's). Dit moet ook in het protocol en de patiënteninformatiebrief worden opgenomen.

De commissie ontvangt graag een reactie op bovenstaande punten, waarbij relevante informatie tevens wordt verwerkt in de diverse documenten.

Belasting

Het risico en de belasting van het onderzoek is de MDCT scan. Dit moet opwegen tegen het belang van de uitkomst van de scan. Het oordeel hierover hangt af van wat daarover bekend is. De commissie is van mening dat de wetenschappelijke relevantie de belasting voor de proefpersoon rechtvaardigt.

9. Toevalsbevindingen zijn een secundaire uitkomstmaat. De commissie verneemt graag met welke mogelijke toevalsbevindingen rekening moet worden gehouden. Zij ziet dit tevens graag verwerkt in het protocol en de patiënteninformatiebrief. Graag ook in het protocol opnemen hoe men hier in de praktijk mee zal omgaan.

Ten aanzien van de documenten heeft de commissie de volgende opmerkingen:

Algemeen

Opgemerkt wordt dat diverse verplichte documenten als bijlage bij het protocol zijn opgenomen. Graag separaat indienen voorzien van datum en versienummer, nu de documenten vaak los van elkaar worden aangepast naar verloop van tijd.

B1. ABR-formulier versie 01 d.d. 20-10-2011

1. Vraag C4: de commissie verzoekt de relatief geringe stralenbelasting nader te specificeren en te vergelijken met bijv. röntgenfoto e.a.; zie ook het protocol, pag. 7, 2^{e} alinea.
2. Vraag C14: invasieve metingen: de commissie merkt op dat de CT scanning wisselend als minimaal invasief en non-invasief wordt beschreven. Zij verzoekt u dit eenduidig te beschrijven als minimaal invasief.
3. Vraag C23: De commissie vraagt zich af of de beoogde 300 patiënten in een jaar geïncludeerd kunnen zijn; zie ook het protocol, pag. 4, kopje 'Methods'. De commissie ontvangt hier graag een toelichting op.
4. Vraag D5a/b: betekent hartfrequentie ≥ 70/min echt exclusie of als dit na metoprolol i.v. nog bestaat? De commissie verzoekt u dit beter te beschrijven.
5. De commissie vraagt zich af of pacemaker ritme een exclusie criterium is. Zo ja, dan ziet zij dit graag vermeld in de diverse documenten, waaronder appendix 8 van het protocol en het ABR-formulier.
6. Vraag E5: de commissie merkt op dat patiënten ook op eventueel andere dan cardiale aandoeningen worden getest.
7. Vraag E6/9: de commissie is van mening dat de stralenbelasting 3 mS per CT meer is dan de jaarbelasting t.g.v. achtergrondstraling.

8. Vraag G: hoe zit het met de financiering? Vragen G1 tot en met G3 zijn wat vreemd ingevuld. De commissie verzoekt vraag G1 te wijzigen in de 1e geldstroom, nu de factuur hierop gebaseerd zal zijn.
9. Vraag K, samenvatting ('Achtergrond van studie' versus 'Background of the study'): 'non-invasief' versus 'minimally invasive'. Graag eenduidig beschrijven. Zie ook de opmerking van de commissie bij vraag C14 en het protocol, opmerking 2.
10. Vraag K1: er wordt wisselend gesproken over SCORE risk chart en Heart Score. (Hier wordt waarschijnlijk steeds bedoeld: SCORE = (Systematic Coronary Evaluation). Graag goed beschrijven, ook in het protocol. De vraag is ook of de Framingham scores niet moeten worden meegenomen. Graag deze punten eveneens verwerken in het protocol. Zie ook pag. 4 van het protocol, kopje 'objectives'. De commissie ontvangt hierop graag een toelichting.

B2. lokaal addendum, ondertekend d.d. 24-10-2011

1. Wordt GFR bepaald bij de nierfunctie bepaling? Dit moet in het formulier worden vermeld. Graag ook eenduidig in het protocol vermelden.

C1. onderzoeksprotocol, versie 10 d.d. 18-10-2011

1. Appendix 14 is een brief met informatie. Wordt het resultaat van het onderzoek alleen op deze wijze aan de proefpersoon medegedeeld?
2. Pagina 4: 'Non-invasief' versus 'minimally invasive': graag eenduidig beschrijven als minimaal invasief. Zie ook vraag 2 en 9 van de commissie m.b.t. het ABR-formulier.
3. Pagina 7, alinea 2: beschreven wordt dat de stralen belasting 3 mS is, dit is de dosering die men in Nederland in 1,5 jaar ontvangt als achtergrondstraling in de buitenlucht. Zoals in het protocol beschreven wordt de indruk gewekt dat dit een lage stralenbelasting is ('very low radiation dose'). Graag correct omschrijven.
4. Pagina 7, alinea 1.3: zie discussie SCORE/Heart Score, vraag 10 m.b.t. het ABR-formulier.
5. Pagina 12: als bijwerking van het toedienen van metoprolol i.v. en nitroglycerine subliguaal kan een bloeddrukdaling optreden. Dit dient in het protocol en de patiënteninformatiebrief te worden opgenomen.
6. Appendix 1, pag. 3: hier staat dat de onwil informatie te ontvangen omtrent eventueel andere, niet cardiale afwijkingen bij de CT scan een exclusiecriterium is. In Appendix 4 en elders moeten de exclusie criteria worden geüniformeerd.
7. Eigenlijk zouden alleen patiënten met een intermediair risico geïncludeerd moeten worden. De in- en exclusie criteria dienen hierop te worden aangepast.
8. Appendix 5, kopje 'algemeen': de commissie verzoekt u deze pagina waar nodig aan te passen. Zie bijv. vraag 1.02, indien 'nee' ga naar 1.03; dit moet zijn 1.04. En zo verder.

E1/2 schriftelijk informatie voor de proefpersonen en/of hun wettelijk vertegenwoordigers, inclusief bijbehorende toestemmingsverklaring(en), versienummer en datum NA

1. Appendix 1, pag. 3: hier wordt een mogelijke bloeddrukdaling na toedienen van metroprolol i.v. en nitroglycerine sublinguaal niet gemeld. De commissie verzoekt dit op te nemen in de brief.
2. De brief is niet conform het format van de CCMO, graag aanpassen met bijbehorende kopjes etc., zodat de brief leesbaarder wordt.
3. De commissie merkt op dat er op de eerste pagina wordt vermeld dat er ook (mannelijke) sporters afkomstig van de sportmedische afdelingen van het UMC Utrecht worden benaderd. De commissie verneemt graag hoe deze patiënten worden benaderd. Indien deze worden benaderd in het UMCU, dan ziet de commissie dit graag terug in het ABR-formulier en het protocol.
4. Tevens ziet de commissie graag vermeld hoe wordt omgegaan met toevalsbevindingen voor wat betreft de behandeling en door wie dit wordt gedaan. De toestemmingsbrief verwijst naar een datum die niet in de brief is vermeld. Graag uniformeren.
5. De patiënteninformatiebrief graag separaat van het protocol indienen en voorzien van datum en versienummer.

Verenigde
Commissies
Mensgebonden
Onderzoek

VCMO

I3. het cv van de hoofdonderzoeker dr. Mosterd, cardioloog Meander Medisch Centrum d.d. NA

1. Uit de publicatielijst is op te maken dat het hier het cv van dr. Mosterd betreft, echter staat zijn naam niet vermeld op het cv. De commissie verzoekt dit aan te passen.

Conclusie
De commissie ontvangt graag antwoord op de gestelde vragen en de aangepaste documenten alvorens zij een beslissing neemt over de deugdelijkheid van het protocol. Tot die tijd wordt de toetsingstermijn opgeschort.

Bovenstaand commentaar is onder voorbehoud van de formele vaststelling van de notulen door de commissie tijdens de eerstvolgende vergadering.

Voor het gemak van de commissie wordt u vriendelijk verzocht om:
- de vragen van de commissie puntsgewijs te beantwoorden;
- de aangepaste documenten te voorzien van een nieuwe datum en versienummer;
- de gewijzigde tekst in de documenten cursief weer te geven en weglatingen door te strepen;
- één "opgeschoond" exemplaar van de gewijzigde documenten bij te voegen.

Bij wijzigingen in het ABR-formulier dient de aangepaste versie opnieuw ondertekend te worden.

De commissie ontvangt uw antwoord graag binnen 3 weken na dagtekening van deze brief (antwoordbrief 1x en bijlagen in 7-voud daarbij). De beoordelingstermijn wordt opgeschort tot het moment dat wij uw reactie volledig hebben ontvangen

Ik verwacht u hiermee voldoende te hebben geïnformeerd.

Met vriendelijke groet,

i/o

Mw. mr. S. de Weerd, ambtelijk secretaris

Namens mw. dr. V.H.M. Deneer
Plv. voorzitter Verenigde Commissies Mensgebonden Onderzoek (VCMO)
