# Supplementary material for: Rationale and design of the Measuring Athlete’s Risk of Cardiovascular events (MARC) study: The role of coronary CT in the cardiovascular evaluation of middle-aged sportsmen
Source: Neth Heart J. 2014 Nov 20;23(2):133–8. doi: 10.1007/s12471-014-0630-0 (PMC4315792; doi:10.1007/s12471-014-0630-0)
Supplement: Supplementary file 2 — (PDF 136 kb) [file 12471_2014_630_MOESM2_ESM.pdf]

Verenigde
Commissies
Mensgebonden
Onderzoek

VCMO

BESLUIT
Primaire beoordeling

NL nummer	NL38234.100.11	VCMO nr.	R-11.44M
Titel onderzoek	Measuring Athlete's Risk of Cardiovascular events		

Contactgegevens: dr. A. Mosterd, cardioloog Meander Medisch Centrum, postbus 1502, 3800 BM Amersfoort
Verrichter: Meander Medisch Centrum te Amersfoort

Besluit
De Verenigde Commissies Mensgebonden Onderzoek (VCMO) heeft zich, op grond van artikel 2, tweede lid, sub a van de *Wet medisch wetenschappelijk onderzoek met mensen* (WMO), beraden over bovenstaand onderzoeksdossier.

De commissie oordeelt positief over de uitvoering van het onderzoek in het volgende centrum
- Meander Medisch Centrum te Amersfoort, hoofdonderzoeker dr. A. Mosterd

Documenten
Het oordeel is gebaseerd op de documenten die in bijlage 1 zijn vermeld.

Achtergrond
Op 20-10-2011 is het onderzoeksdossier ter beoordeling bij de VCMO ingediend. Na ontvangst van het ontbrekende document op 24-10-2011, is het dossier in behandeling genomen. Het onderzoeksdossier is besproken in de vergaderingen van 07-11-2011 en 02-01-2012. Op 20-01-2012 is de ondertekende versie van het lokaal addendum ontvangen. Zie bijlage 2 voor de samenstelling van de commissie / aanwezige leden op de vergadering van 07-11-2011.

Overwegingen
De VCMO is van oordeel dat aan de voorwaarden in artikel 3 van de WMO is voldaan. De belangrijkste vragen waren de klinische betekenis van de studie, de vraagstelling en de mogelijke toevalsbevindingen. Daarnaast had de commissie opmerkingen over de beperkingen van de studie m.b.t. het ontbreken van de toegevoegde waarde van screening bij laag- en hoogrisico patiënten, potentieel nadelige gevolgen ten gevolge van vals positieve testen en blootstelling aan straling en het gebrek aan bewijs van het nut van medicamenteuze behandeling van asymptomatische patiënten op klinisch beloop.

De belangrijkste argumenten van de commissie om over te gaan tot een positief besluit zijn o.a. dat de vragen genoegzaam zijn beantwoord en de documenten correct zijn aangepast

De commissie heeft de inhoud van de lokale uitvoerbaarheidsverklaring van de Raad van Bestuur/Directie van de deelnemende instelling bekeken. Zij heeft geconstateerd dat is voldaan aan de voorwaarden in artikel 3, onderdeel e en j, van de WMO.

Verzekeringen
De VCMO heeft geconstateerd dat is voldaan aan de verzekeringsplicht. Er is een proefpersonenverzekering afgesloten zoals bepaald in artikel 7, eerste lid van de WMO en zoals nader uitgewerkt in het *Besluit verplichte verzekering bij medisch-wetenschappelijk onderzoek met mensen (Besluit van 23 juni 2003, Stb. 2003, 266).*

Het onderzoek valt onder de proefpersonenverzekering van het Meander Medisch Centrum.

Verenigde
Commissies
Mensgebonden
Onderzoek

VCMO

De commissie heeft geconstateerd dat een aansprakelijkheidsverzekering is afgesloten zoals bepaald in artikel 7, zesde lid van de WMO.

Ten slotte wijst de VCMO u op de voorwaarden en verplichtingen die in bijlage 3 zijn vermeld.

Met vriendelijke groet,

Mw. mr. S. de Weerd, ambtelijk secretaris

Namens dr. A. Hovestadt
voorzitter Verenigde Commissies Mensgebonden Onderzoek (VCMO)

Nieuwegein, 20-01-2012

Beroepsprocedure
Tegen dit besluit kan een belanghebbende op grond van artikel 23 WMO binnen zes weken na de dag waarop het besluit is bekend gemaakt, administratief beroep instellen bij de Centrale Commissie Mensgebonden Onderzoek (CCMO). Het beroepschrift dient u te adresseren aan CCMO, Postbus 16302, 2500 BH Den Haag.

Verenigde
Commissies
Mensgebonden
Onderzoek

VCMO

Bijlage 1

Documenten

A. Brieven

A1. aanbiedingsbrief d.d. 20-10-2011, ontvangen d.d. 20-10-2011

A1. correspondentie: vraagbrief VCMO d.d. 25-11-2011 met kenmerk V.11.416, antwoordbrief indiener d.d. 19-12-2011, ontvangen d.d. 20-12-2011

A1. correspondentie: vraagmail VCMO d.d. 13-01-2012, antwoordbrief indiener, ontvangen d.d. 20-01-2012

B. Formulieren

B1. ABR-formulier versie 03 d.d. 19-12-2011

B2. lokaal addendum, ondertekend d.d. 19-01-2012, ontvangen d.d. 20-01-2012

C. Onderzoeksprotocol en eventuele protocol amendementen

C1. onderzoeksprotocol, versie 2 d.d. 15-12-2011

C1. appendix 4 – in-/exclusiecriteria, versie 2 d.d. 15-12-2011

C1. appendix 9 – CT Protocol and Case Record Form, versienr. en datum NA

C1. appendix 11 – Costs, versienr. en datum NA

C1. appendix 15 – Cutoff Values for CACS, versienr. en datum NA

E. Informatie voor proefpersonen

E1/2. schriftelijk informatie voor de proefpersonen en/of hun wettelijk vertegenwoordigers, inclusief bijbehorende toestemmingsverklaring, versie 2 d.d. 15-12-2011

E4. letter and advice for participant, versie 2 d.d. 15-12-2012

F. Te gebruiken vragenlijsten, patiëntendagboekje, patiëntenkaart, etc.

F1. vragenlijsten:

- MARC onderzoek, versie 2 d.d. 15-12-2011
- Appendix 10 – Vragenlijst evaluatie MARC-onderzoek, versienr. en datum NA

F4. overig:

- Appendix 8 - CRF ECG-evaluation form, versie 2 d.d. 15-12-2011
- Individual summary of results per participant, versie 2 d.d. 15-12-2011
- Appendix 6 - Lichamelijk onderzoek & aanvullend onderzoek, versienr. en datum NA
- Appendix 7 - Lifetime exercise and sports activity questionnaire, versienr. en datum NA
- Appendix 12 - Checklist onderzoekers, versienr. en datum NA

G. Informatie over de verzekering

G1. WMO proefpersonenverzekering van het Meander Medisch Centrum, polisnummer MR086/073778/WMO van verzekeringsmaatschappij MediRisk d.d. 11-02-2009

G2. bewijs dekking aansprakelijkheid Meander Medisch Centrum, polisnummer MR086/073778.99 van verzekeringsmaatschappij MediRisk d.d. 03-02-2011

H. CV's

H1. het cv van de onafhankelijk arts dr. A.W.J. van 't Hof d.d. NA

I. Informatie per deelnemend centrum in Nederland

I3. het cv van de hoofdonderzoeker dr. A. Mosterd, cardioloog Meander Medisch Centrum, versie 2 d.d. 15-12-2011

K. Overige relevante documenten:

K4. wetenschappelijke publicaties over eerder vergelijkbaar onderzoek, ontvangen d.d. 20-10-2011

Bijlage 2

Samenstelling VCMO
De volgende leden waren aanwezig tijdens de commissievergadering van 07-11-2011:

mw. dr. V.H.M. Deneer, klinisch farmacoloog (plv. voorzitter)
mw. dr. S.K. Klein, internist
dhr. dr. E.F.D. Wever, cardioloog
dhr. dr. R.M.J. Wesselink, anesthesioloog/intensivist
mw. dr. G van Thiel, ethica
mw. mr. P.J.F. van Paridon, jurist
dhr. dr. E.M.W. van de Garde, ziekenhuisapotheker
dhr. dr. C.S.P.M. Uiterwaal, methodoloog
mw. L. Lim, patiëntenvertegenwoordiger
mw. I. Hofstede, verpleegkundige

Verenigde
Commissies
Mensgebonden
Onderzoek

VCMO

Bijlage 3

Voorwaarden en verplichtingen*

- **geldigheid oordeel**:
  het positieve oordeel verliest zijn geldigheid als met het uitvoeren van het onderzoek niet is begonnen binnen een jaar nadat dit besluit is genomen;

- **amendementen**:
  amendementen dienen ter beoordeling aan de VCMO te worden voorgelegd;

- **startdatum onderzoek**:
  de VCMO dient op de hoogte te worden gesteld van de definitieve startdatum van het onderzoek. Dat is de datum waarop de inclusie van de eerste patiënt heft plaatsgevonden;

- **voortgangsrapportage**:
  één jaar na datum oordeel, en ieder jaar daaropvolgend, dient de VCMO op de hoogte te worden gebracht van de voortgang van de studie middels het formulier 'Voortgangsrapportage';

- **geldigheid verzekering**:
  in het geval het verzekeringscertificaat tijdens de voortgang van het onderzoek zijn geldigheid verliest, dient aan de VCMO tijdig een afschrift van een nieuw geldig certificaat te worden toegestuurd;

- **melding artikel 10**:
  indien het onderzoek een verloop neemt dat in noemenswaardige mate voor de proefpersoon ongunstiger is dan in het onderzoeksdossier is voorzien, moet daarvan terstond mededeling worden gedaan aan de VCMO met een verzoek tot nader oordeel;

- **melding SAE's**
  SAE's dienen aan de VCMO gemeld te worden

- **melding (voortijdige) beëindiging**:
  (voortijdige) beëindiging van het onderzoek dient, met redenen omkleed, te worden gemeld aan de VCMO.

- **eindrapportage**:
  de VCMO dient op de hoogte te worden gebracht van de resultaten van het onderzoek middels een eindrapport.

* Termijnen en overige uitleg ten aanzien van de indiening van de verschillende documenten aan de VCMO vindt u in ToetsingOnline en op de website van de CCMO bij het standaard onderzoeksdossier en de toelichting daarop.
